# Investigation of intra-fractionated range guided adaptive proton therapy (RGAPT): II. Range-shift compensated on-line treatment adaptation and verification

**DOI:** 10.1088/1361-6560/ad56f2

**Published:** 2024-07-17

**Authors:** Mingli Chen, Dongxu Yang, Xiaorong R Zhu, Lin Ma, David R Grosshans, Yiping Shao, Weiguo Lu

**Affiliations:** 1 Department of Radiation Oncology, University of Texas Southwestern Medical Center, Dallas, TX 75390, United States of America; 2 Department of Radiation Physics, University of Texas MD Anderson Cancer Center, Houston, TX 77030, United States of America; 3 Department of Radiation Oncology, University of Texas MD Anderson Cancer Center, Houston, TX 77030, United States of America; 4 Co-first authors.

**Keywords:** proton beam range, range guided adaptive proton therapy, RGAPT

## Abstract

We previously proposed range-guided adaptive proton therapy (RGAPT) that uses mid-range treatment beams as probing beams and intra-fractionated range measurements for online adaptation. In this work, we demonstrated experimental verification and reported the dosimetric accuracy for RGAPT. A STEEV phantom was used for the experiments, and a 3 × 3 × 3 cm^3^ cube inside the phantom was assigned to be the treatment target. We simulated three online range shift scenarios: reference, overshoot, and undershoot, by placing upstream Lucite sheets, 4, 0, and 8 that corresponded to changes of 0, 6.8, and −6.8 mm, respectively, in water-equivalent path length. The reference treatment plan was to deliver single-field uniform target doses in pencil beam scanning mode and generated on the Eclipse treatment planning system. Different numbers of mid-range layers, including single, three, and five layers, were selected as probing beams to evaluate beam range (BR) measurement accuracy in positron emission tomography (PET). Online plans were modified to adapt to BR shifts and compensate for probing beam doses. In contrast, non-adaptive plans were also delivered and compared to adaptive plans by film measurements. The mid-range probing beams of three (5.55MU) and five layers (8.71MU) yielded accurate range shift measurements in 60 s of PET acquisition with uncertainty of 0.5 mm while the single-layer probing (1.65MU) was not sufficient for measurements. The adaptive plans achieved an average gamma (2%/2 mm) passing rate of 95%. In contrast, the non-adaptive plans only had an average passing rate of 69%. RGAPT planning and delivery are feasible and verified by the experiments. The probing beam delivery, range measurements, and adaptive planning and delivery added a small increase in treatment delivery workflow time but resulted in substantial dose improvement. The three-layer mid-range probing was most suitable considering the balance of high range measurement accuracy and the low number of probing beam layers.

## 1. Introduction

Proton beams offer dosimetric advantages with low entrance dose, peak dose before the end of range, and a sharp distal fall-off, allowing for the same target dose while lowering integral dose compared to photon beams (Paganetti and Bortfeld 2005). However, the advantage can create a double-edged sword, as even a small shift in the beam spot position of a few millimeters can result in a large dose difference, making proton beams susceptible to beam range (BR) uncertainty (Verburg 2015).

There are various sources of BR uncertainty, attributable to anatomical changes, patient setup positioning, CT-based stopping power ratio calculation, machine delivery system (Paganetti *et al* 2021). Certain sources of uncertainty are common and have a greater impact, such as anatomical changes, while others have a relatively minor impact, such as uncertainty in the machine delivery system. This is because the machine is routinely monitored and proactively corrected as part of clinical quality assurance in a clinical environment.

Addressing the uncertainty is critical to exploit the distal gradient and has been an area of active research in proton therapy (Paganetti *et al* 2021), from improving precision, such as range determination (Yang *et al* 2012), range measurements (Knopf and Lomax 2013, Parodi and Polf 2018, Zhong *et al* 2020), and plan adaptation (Zhang *et al* 2011), to improving robustness, such as robust planning and delivery by embracing the uncertainty (Unkelbach *et al* 2009, Fredriksson *et al* 2011). Intuitively, robust planning and delivery considers various delivery scenarios in the treatment planning, reduces the weights of beams that abut critical organs through optimization, but may provide an average plan or plan for the worst-case scenario, which however would not take full advantage of proton beams. On the other hand, improving precision requires further range accuracy and efficient adaptive planning, both of which are highly demanding on hardware and software capabilities.

**Figure 1.**
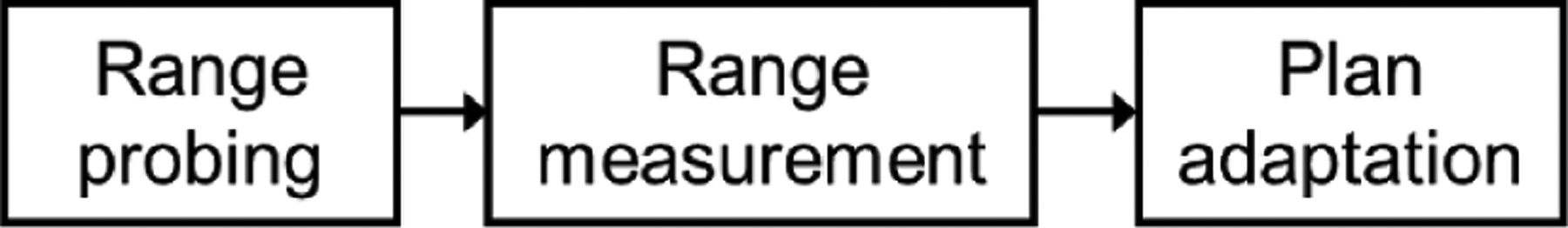
A simplified chart of online RGAPT.

We previously proposed a range-guided adaptive proton therapy (RGAPT) strategy utilizing planned mid-range spots to probe BRs and adapting the plan following range measurements (Chen *et al* 2018). Figure 1 outlines the three major steps of RGAPT. Though a simple strategy, RGAPT entails multiple advantages: treatment beams offer stronger signals for range measurements than imaging beams (Shao *et al* 2014); mid-range spots likely remain in the target without causing extra radiation to the surrounding normal tissues given the range uncertainty; plan adaptation can account for both BR shift and probing beam dose due to high degrees of freedom in proton planning. We have subsequently developed an in vivo online positron emission tomography (PET) system (Yang) to measure the probing BR and conducted an initial experiment to test the RGAPT planning and accuracy.

The purpose of this study has two folds: demonstrating the feasibility of RGAPT through experiments and addressing the precision of RGAPT. In this part II of the paper, we will describe the adaptive planning and the achieved dosimetric accuracy in the experiment. The details and specifications of the in-house development and evaluation of the on-line PET imaging for intra-fractionated proton range measurement are reported in part I of the paper.

## 2. Method

Below we give a brief account of our RGAPT systems. Then, we describe our experiments, including treatment planning, beam delivery, film measurements, and data analysis.

### 2.1. The RGAPT system

The RGAPT workflow is briefly described here for the reader’s convenience. The detail can be found in our previous publication (Chen *et al* 2018). From the treatment plan, a small number of mid-range treatment spots are selected as the probing beam for online range measurement. The probing beam is delivered prior to other spots. Then, the range is measured and compared to calculation. The expected BR of probing beam can be calculated according to the treatment plan via Monte Carlo (MC) simulation, while PET measurement provides the activity range of positron emitters. To enable comparison, MC simulations are employed to establish the relationship between AR and BR (Mashayekhi *et al* 2017, Ma 2022). If there is a range shift, the remaining treatment spots are modified through adaptive planning, which also accounts for the delivered probing spots. In this work, we will adopt a simple range compensation strategy. Let *I*_0_ (*R,x,y,ϕ*) denote the original plan, where *I* denotes the spot intensity in monitor units (MU), *R* the spot range, *x* and *y* the transverse position, and *ϕ* the beam angle. Note that the spot range in water equivalent path length (WEPL) and energy have a one-to-one correspondence. To simplify the adaptive plan formulation, the spot intensity is expressed as a function of the spot range instead of a function of the energy. Suppose the online measured spot range *R*_1_ and the planned spot range *R*_0_ has a difference Δ*R* = *R*_1_ *− R*_0_. The range compensation is implemented for plan adaptation as follows,

(1) $$I_{1}(R,x,y,\phi )=\left\{\begin{array}{c} I_{0}(R+\Delta R,x,y,\phi ),R\neq R_{0}\\ {\left(I_{0}\left(R_{0}+\Delta R,x,y,\phi \right)-I_{0}\left(R_{0},x,y,\phi \right)\right)}_{+},R=R_{0} \end{array}.\right.$$

In equation (1), the already delivered probing beam intensity is subtracted from the plan. Alternatively, plan adaptation can be calculated through optimization (Chen *et al* 2018).

**Figure 2.**
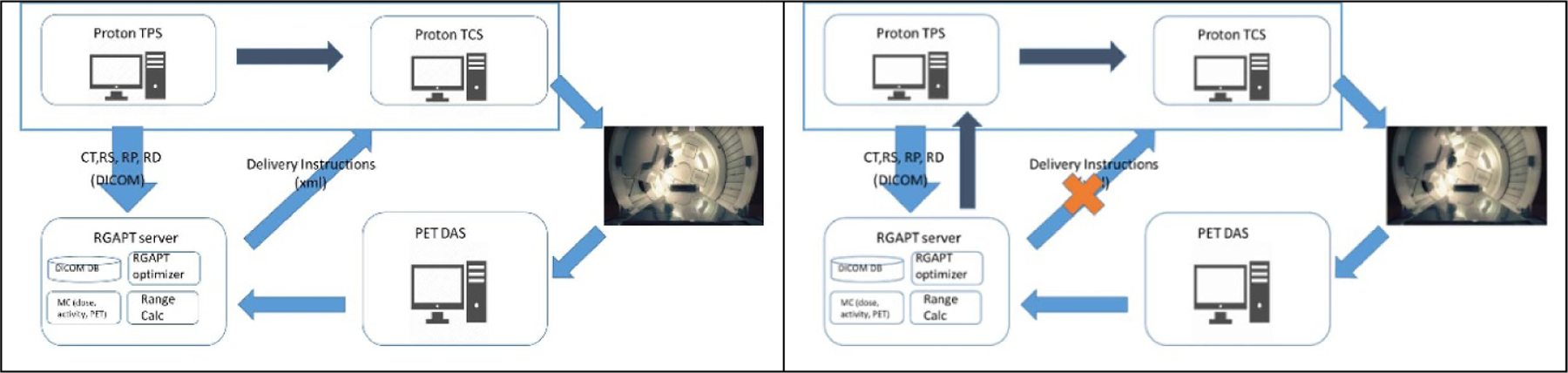
RGAPT system design workflow. Left: ideal workflow. Right: experimental workflow.

The RGAPT system included a server for data management and calculation software and a workflow integration with the treatment planning system (TPS), treatment control system (TCS), and the PET system. In the ideal RGAPT system workflow (figure 2, left side), the patient CT images, RT structures, plans, and doses are retrieved from the TPS. Then, the RGAPT server selects the probing beam and sends the delivery instruction to the TCS. The PET data acquisition system (DAS) measures the positron source positions and sends the results to the server. The server performs range calculation and comparison with measurements, calculates the adaptive treatment plan, and sends the delivery instruction to the proton TCS. However, directly communicating with the TCS is not practical or needed at this stage. For our experimental aims, it suffices to go through the TPS as in figure 2, right side.

### 2.2. Experiments

**Figure 3.**
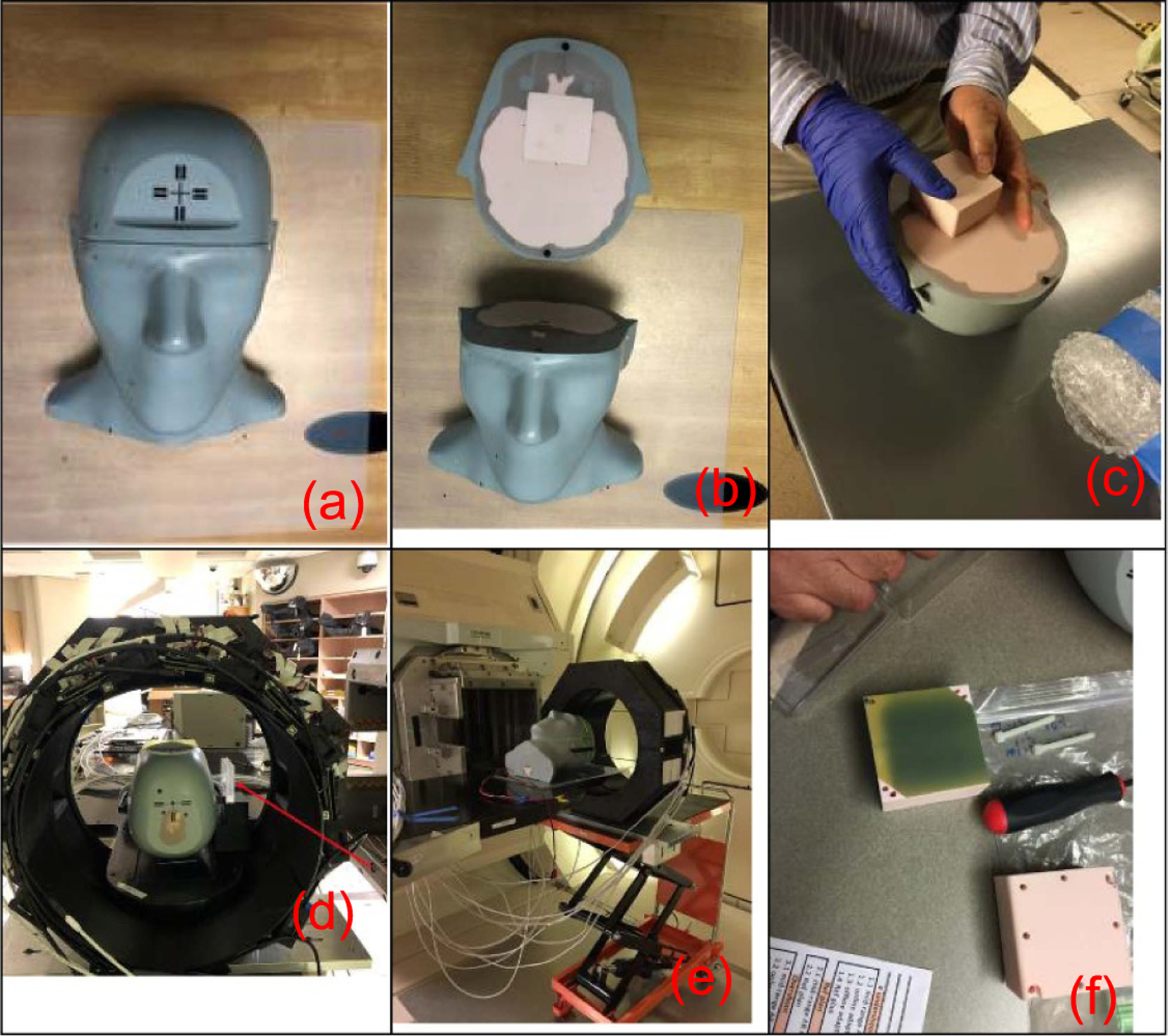
Experimental setup and processes. (a) The STEEV phantom. (b) Opening the top portion of the phantom to insert film. (c) Putting back the cube that sandwiched the film. (d) Illustration of Lucite sheets placement, indicated by the red arrow. (e) Placing the phantom and the PET gantry in the beam delivery position for measuring positron activity during proton beam delivery. (e) Taking out the film for processing after beam delivery and PET acquisition.

The experiments were conducted using the Hitachi proton treatment system (Smith *et al* 2009) at M D Anderson Cancer Center in Houston, Texas. The experimental setup and processes are illustrated in figure 3. A stereotactic end-to-end verification (STEEV) phantom (figure 3(a)) was used for the experiments. The phantom had a 6 × 6 × 6 cm^3^ cube insert (figure 3(b)), and the plan was to deliver single-field uniform dose (SFUD) to the central 3 × 3 × 3 cm^3^ volume of the cube. The reference plan was generated by the Eclipse TPS for the spot scanning delivery mode.

Before the beam delivery, the cube was temporarily removed for film placement and then repositioned back into the phantom (figure 3(c)). The Lucite sheets were placed on the beam path before the phantom (figure 3(d)). Both the STEEV phantom and the PET gantry were positioned in front of the proton delivery snout and aligned by laser alignment before beam delivery (figure 3(e)). PET data were acquired immediately following the delivery of the probing beam. After ~1 min of PET acquisition, we waited ~1 min for reconstruction. The film was removed after beam delivery for film processing (figure 3(f)).

Three range shift scenarios were simulated: reference, overshoot, and undershoot, by placing 4, 0, and 8 Lucite sheets upstream the proton beam, corresponding to range shifts of 0, 6.8, and −6.8 mm, respectively, in WEPL. Different numbers of mid-range probing beam layers were selected: single, three, and five energy layers, to evaluate the range measurement accuracy. Here, an energy layer refers to the collection of spots with the same energy. For each combination of range shift scenario and probing beam selection, an adaptive plan was generated to account for the measured range shift and probing beam dose. If no range shift was detected, the remaining treatment spots were not changed, which was also for non-adaptive plans. To quantify achievable accuracy through plan adaptation, we assumed measured range shifts were accurate for plan adaptation, like conducting unit testing.

The PET imaging setup, data acquisition and processing, image reconstruction, and proton-induced activity range measurement with probing beams are detailed in part I.

### 2.3. Film processing and analysis

**Figure 4.**
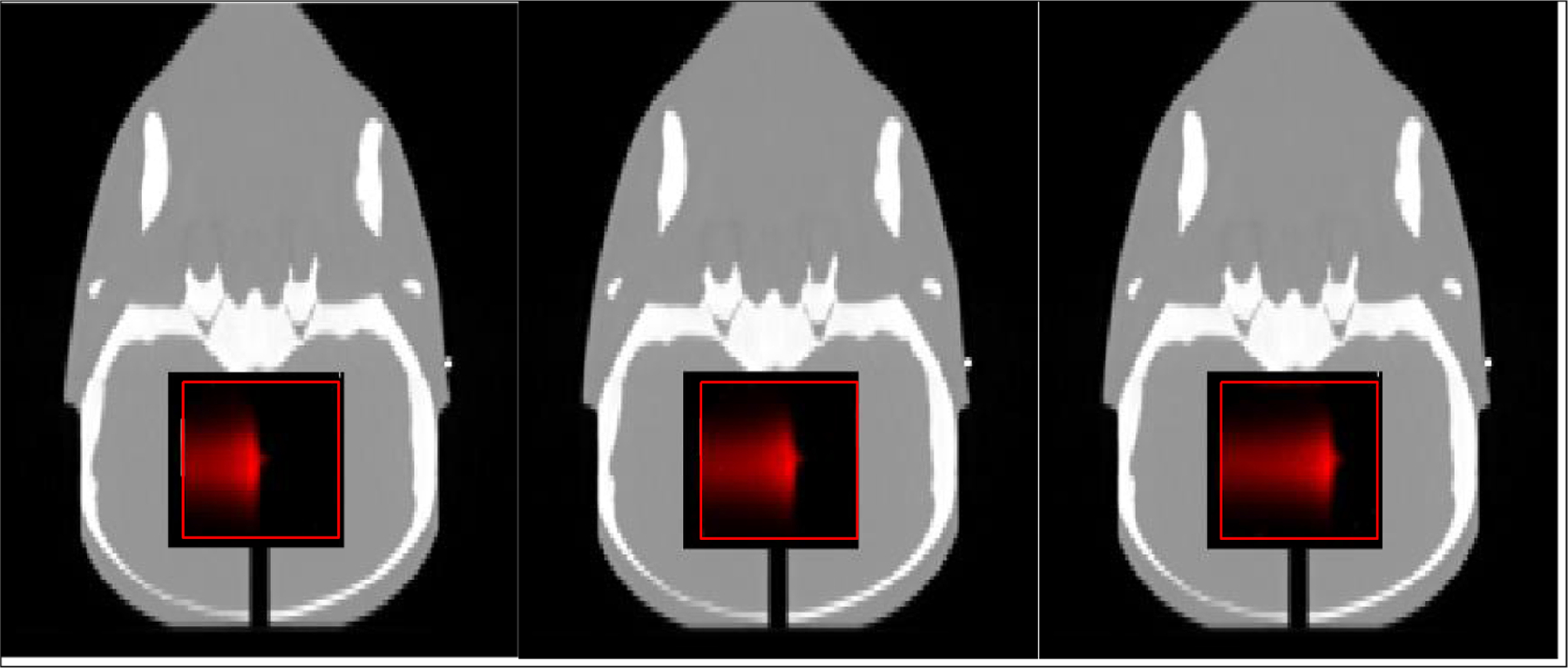
Illustration of parsed, aligned, and rotated films (outlined in red square) overlaid on the STEEV phantom (coronal slice). Left to right: undershoot, reference, overshoot.

The films had dimensions of ~6 × 6 cm^2^, which were cut from regular-sized sheets. The films were scanned in a batch to save time. After batch scanning, we detected film corners, parsed the films, and saved them individually for dose evaluations, including comparison of *R*_50_ and the Gamma Index analysis. Figure 4 illustrates the processed films overlaid on the STEEV phantom.

## 3. Results

### 3.1. Plan

The reference proton plan (single field uniform dose) was generated by the Eclipse TPS and consisted of 22 layers and 1382 spots (see appendix). The layers and spots were recorded in .csv files in decreasing energy from 124 MeV to 95.7 MeV with an average energy resolution of 1.35 MeV (~2 mm). The single layer mid-range probing used layer #11 with 1.65 MU corresponding to radiation dose of 0.0718 Gy to the target. The three-layer probing used layer #10-#12 with 5.55 MU (0.2416 Gy). The five-layer probing used layer #9-#13 with 8.71 MU (0.3791 Gy). The whole plan used a total of 45.95 MU and delivered 2 Gy to the target.

### 3.2. On-line PET measurements

**Figure 5.**
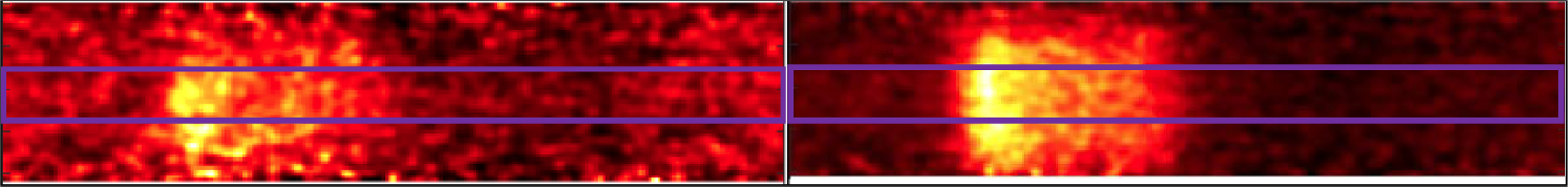
The reconstructed PET images for mid-range probing. Left: a single-layer range probing. Right: a three-layer range probing. The *x*-axis is the beam direction that goes from left to right. The *y*-axis is the longitudinal direction. The beam range is near the center of the image while the bright intensity is around the phantom surface. The rectangles indicate the regions from which we extracted profiles for beam range measurements. The measurement details of the activity range are provided in part I of this two-part work.

Figure 5 shows an image slice of the reconstructed PET image for the probing beams of the single energy layer (left) and three energy layers (right) mid-range spots delivered in the reference scenario. The reconstructed 3D PET image dimensions were 256 × 64 × 64 mm^3^, and the resolutions were 1 × 1 × 1 mm^3^. In the image slices, the *x*-axis is the beam direction that goes from left to right. The *y*-axis is the longitudinal direction. To reduce noise in the BR measurements, we averaged the profiles within a 10 × 10 mm^2^ rectangular cross-section region around the center of spot as indicated in figure 5. For the midrange energies utilized in our study, the spot size was greater than 20 mm (Gillin *et al* 2010). Therefore, these profiles were well within a spot, and their average can be used to measure the spot range.

**Figure 6.**
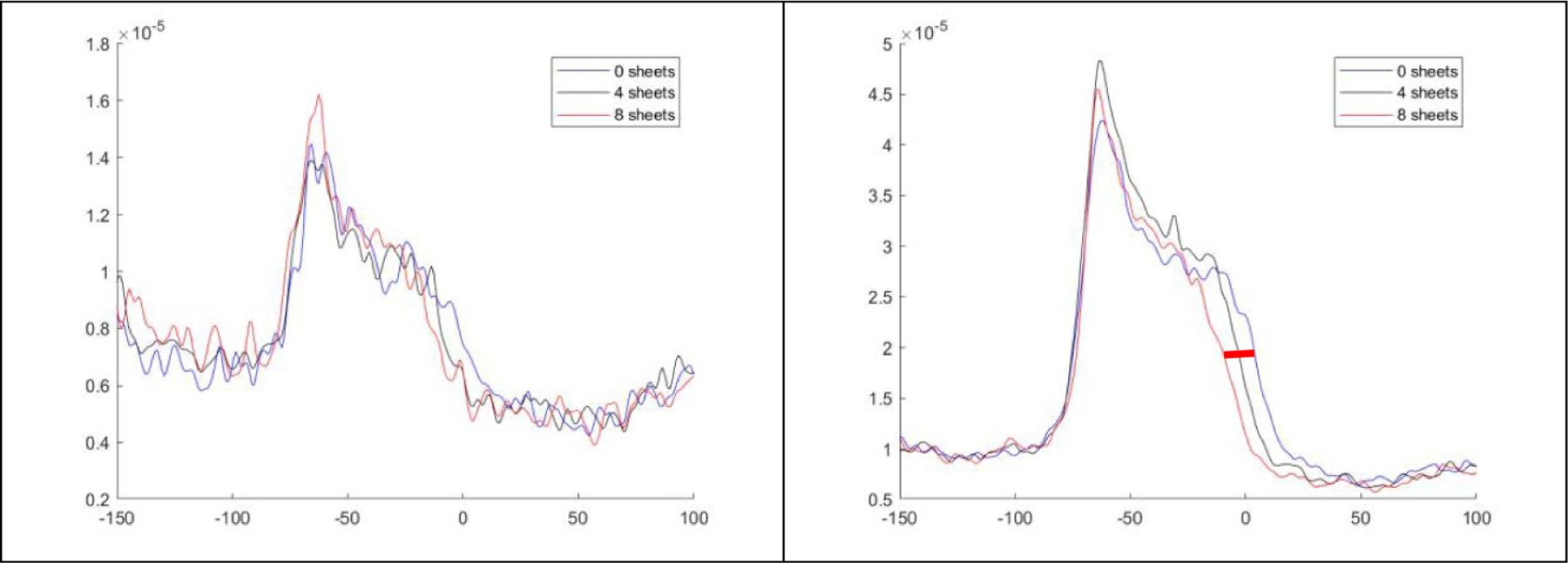
The PET image profiles used for beam range measurement. Left: one layer of probing beams. Right: three layers of probing beams. The red bar indicates the range shift measurements.

Figure 6 shows the average profiles with BR shifts near the center (*x* = 0). The three-layer mid-range probing provided better distinguishable BR shifts than the single-layer. The five-layer probing is not shown as it had similar range shift measurement results to those of the three-layer. Note that the peaks around the phantom surface were likely caused by the surface material but not related to the range measurements.

The PET data acquisition was 1 min as well as the image reconstruction time. We also waited 13 min before the next set of probing beam delivery and data acquisition to minimize the residual radioactivity sources in the phantom.

### 3.3. Film measurements

**Figure 7.**
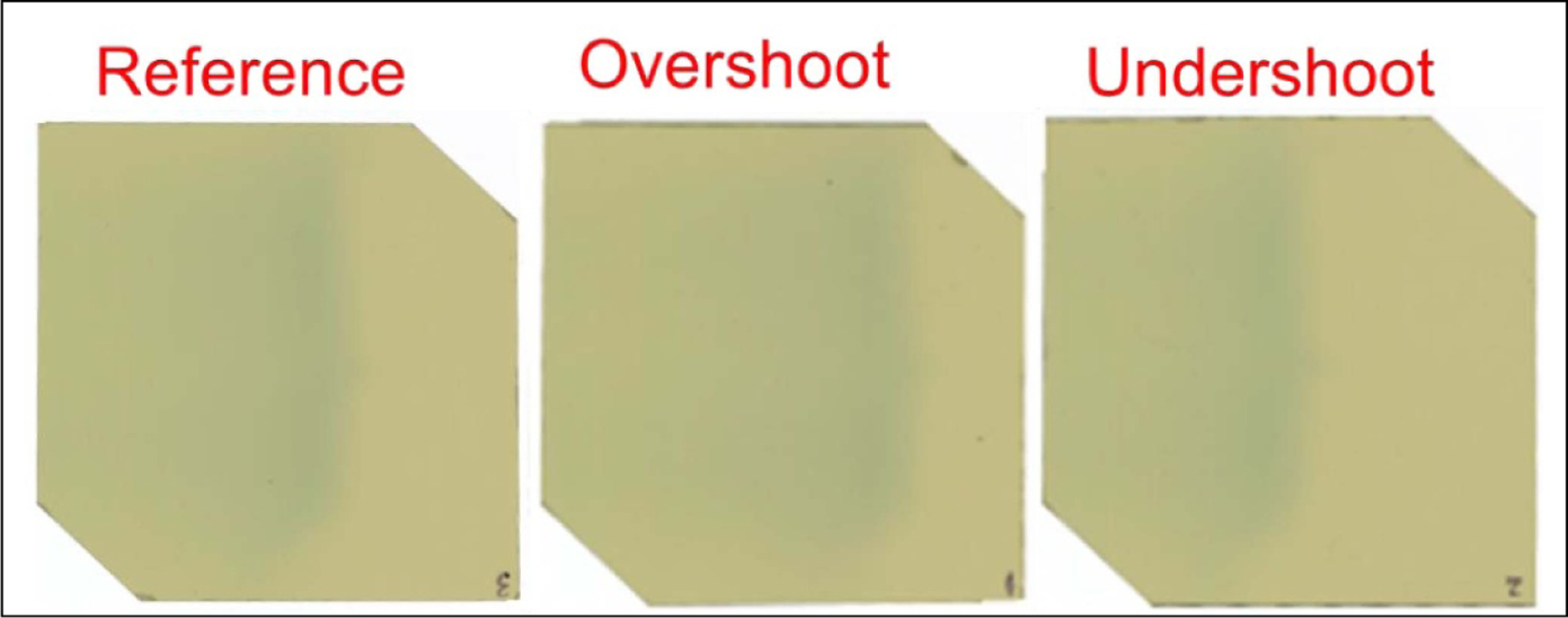
Film measurements of three-layer probing beams for different range shift scenarios.

**Figure 8.**
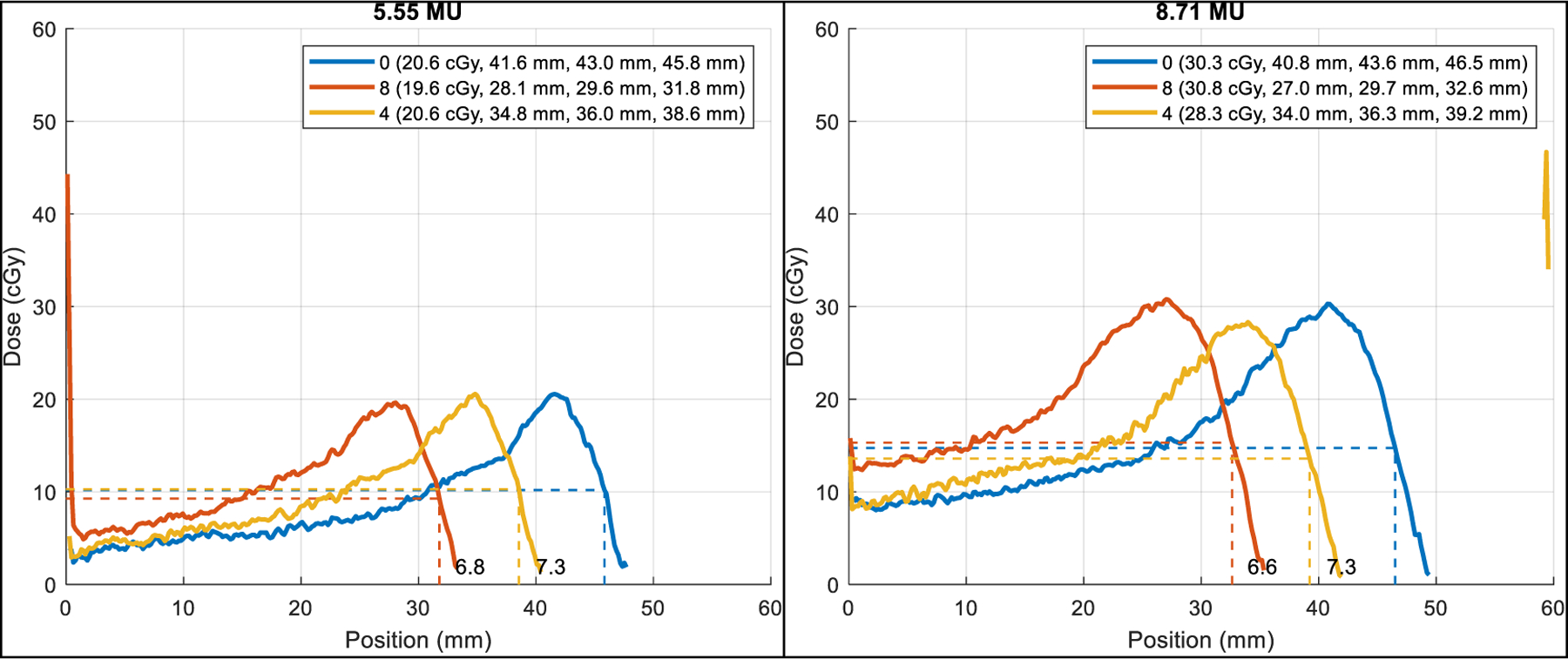
Probing beam dose profiles.

Figure 7 shows the films for the three-layer probing beams delivered to different range shift scenarios with clear shifts at the distal end. Figure 8 shows the converted dose profiles for the three-layer (left) and five-layer (right) probing using a published calibration curve (Casanova Borca *et al* 2013). The single layer probing was out of the calibration range though the raw intensity could still be used to measure the BR. The numerical results (*D*_max_ and *R*_50_) of the profiles are summarized in table 1. However, since film calibration is subject to various factors, including beam energy and types, the dose values presented here are intended for relative comparison. Note that the changes in WEPL were supposed to be 6.8 mm with 0 or 8 sheets of Lucite compared to 4 sheets. However, the removal of Lucite (sheet = 0) led to a larger change in WEPL (ΔWEPL > 6.8 mm). This is likely due to the increased effect of range straggling with higher energy (0 sheets correspond to higher beam energy).

**Figure 9.**
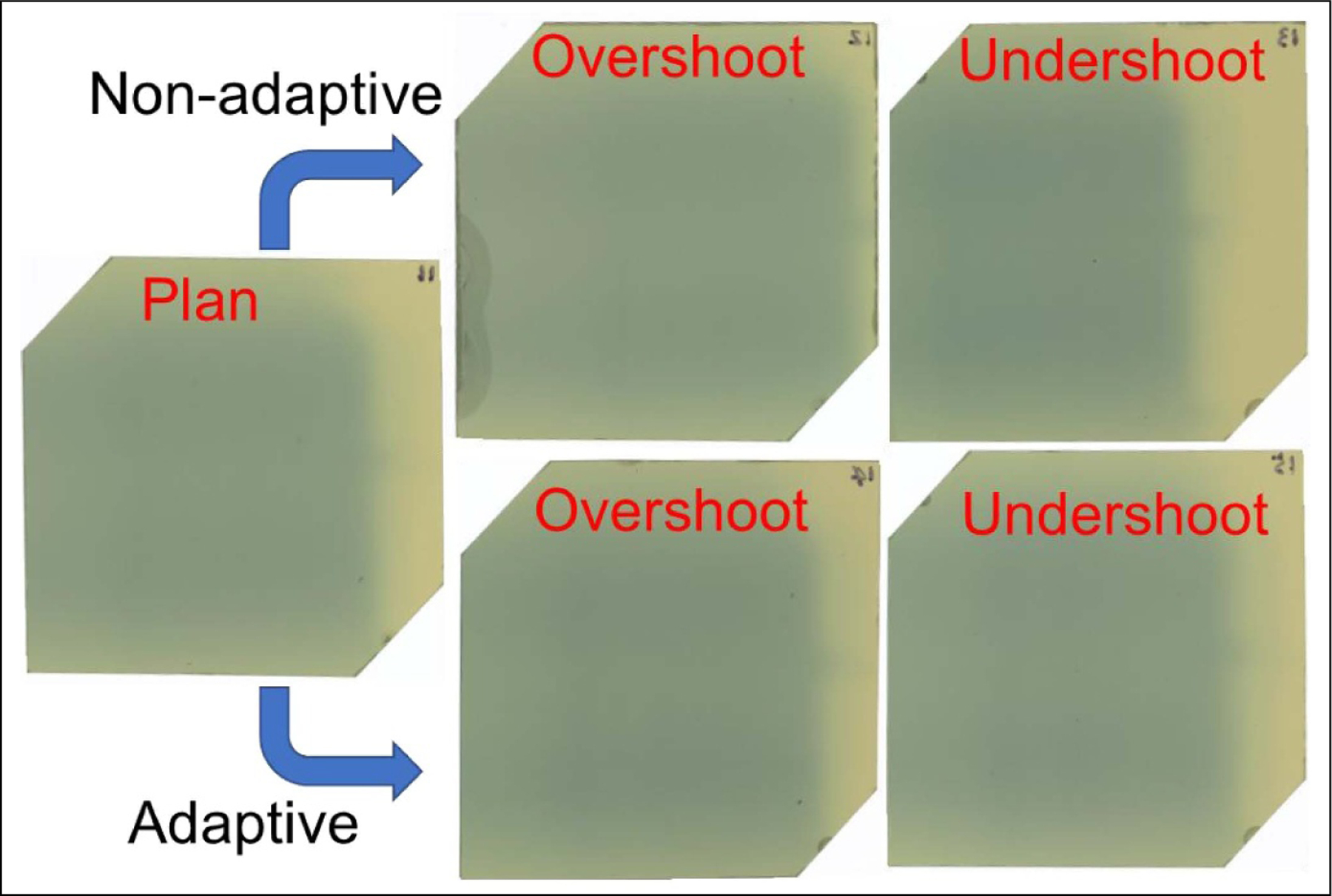
Film measurements for the reference, non-adaptive, and adaptive plans. The online scenarios included overshoot (0 sheets) and undershoot (8 sheets) scenarios.

**Figure 10.**
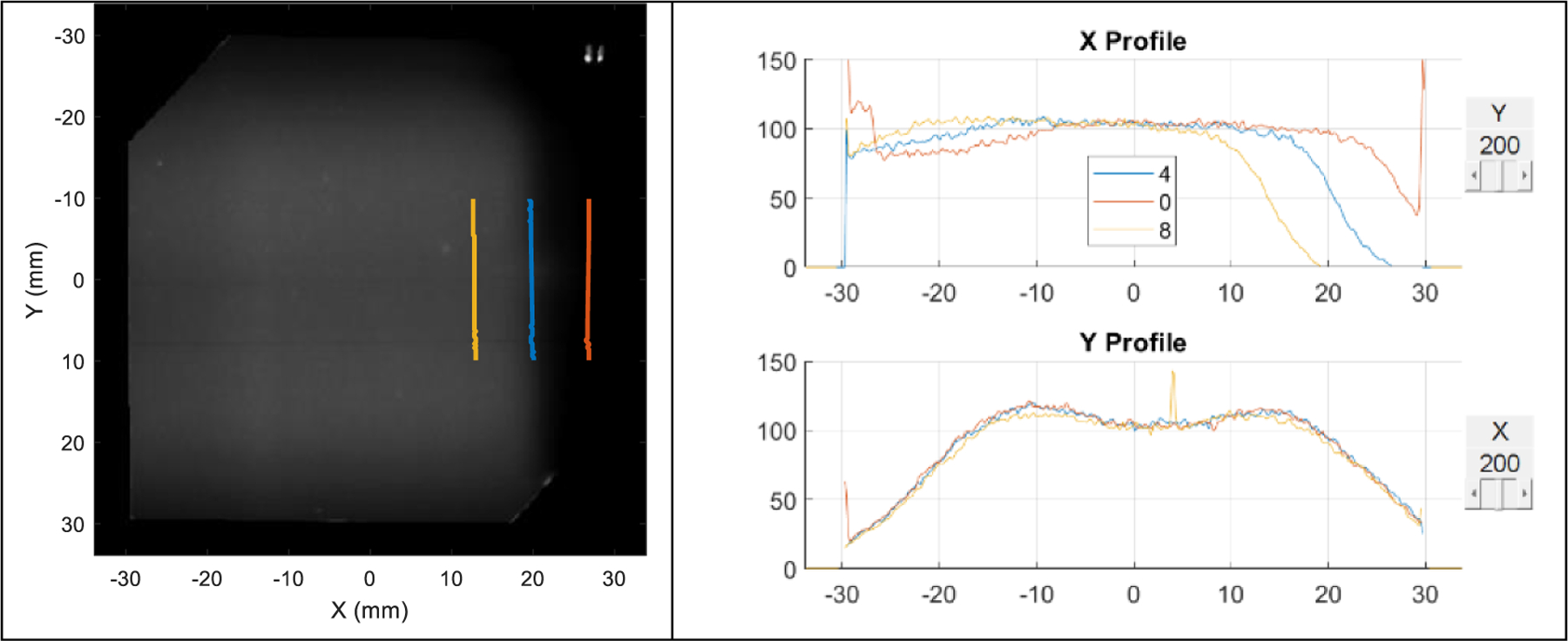
Reference and non-adaptive doses. Left: dose image of the reference plan. The three short line segments around the 20 mm mark indicate the *R*_50_ position of each *x*-profile for *y* ranging between −10 mm and 10 mm for the undershoot (yellow), reference (blue), and overshoot (red) scenarios. Right: the *x*- and *y*- dose profiles of the three scenarios.

**Figure 11.**
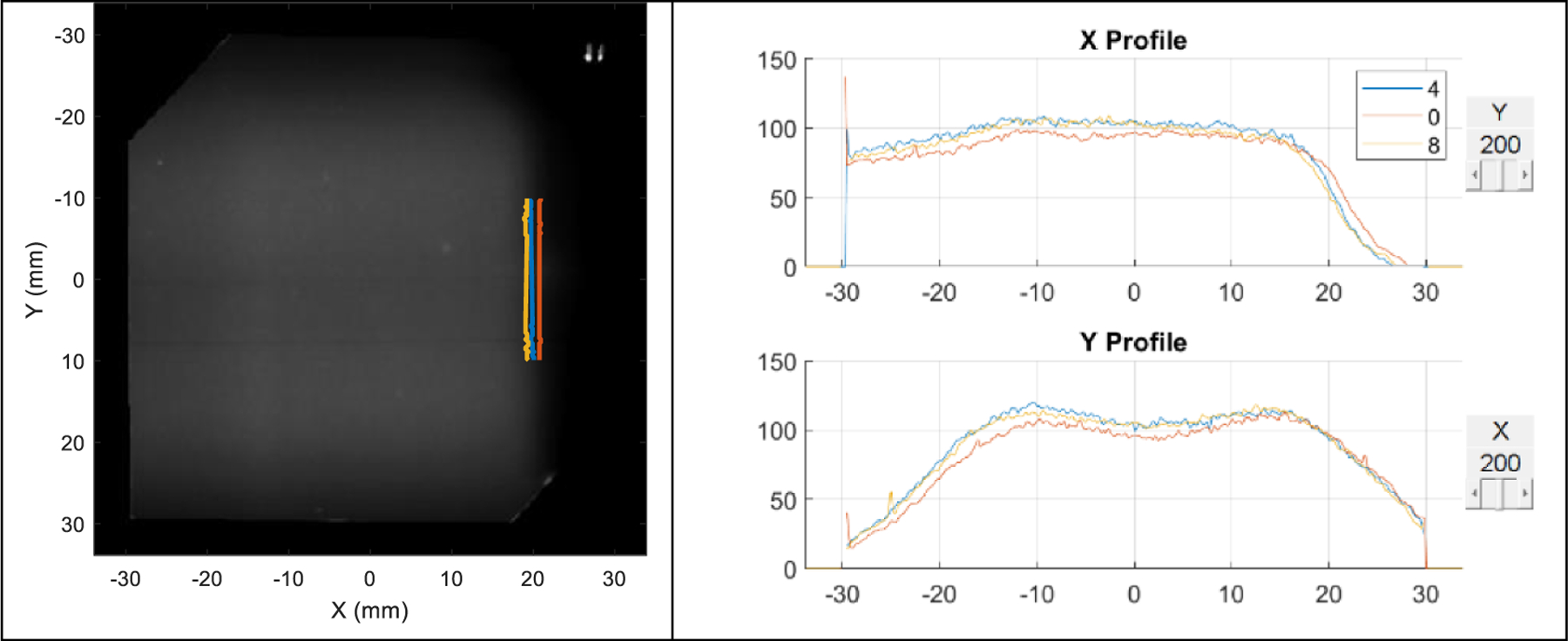
Five-layer midrange and adaptive plans. Left: dose image of the reference plan. The three short line segments around the 20 mm mark indicate the *R*_50_ position of each *x*-profile for *y* ranging between −10 mm and 10 mm of adaptive plans for the undershoot (yellow) and overshoot (red) scenarios. The reference is marked in blue. Right: the *x*- and *y*- dose profiles of the three scenarios.

The plan adaptation was applied to both the overshoot and undershoot scenarios and compared to non-adaptive plans. Figure 9 shows the films of the reference, non-adaptive, and adaptive plans. Without adaptation, the films were clearly different at the distal end for both range shift scenarios compared to the reference, while with adaptation, both cases were corrected. This is also illustrated in the dose image and profiles. Figure 10, left side, shows the *R*_50_’s of non-adaptive plans for overshoot (red) and undershoot (yellow) overlaid on the reference dose with 7 mm from the reference *R*_50_ (blue). Figure 10, right side, shows their dose profiles. Figure 11, left side, shows the *R*_50_’s of adaptive plans for overshoot (red) and undershoot (yellow) overlaid on the reference dose within 1 mm from the reference (blue). Figure 11, right side, shows their dose profiles. Legend 4, 0, and 8 are numbers of sheets, indicating reference, overshoot, and undershoot, respectively. Table 2 shows the gamma index for the non-adaptive plans against the reference, and table 3 are for adaptive plans. Without adaptation, the gamma index (2%, 2 mm) for the overshoot and undershoot was 72% and 66%, respectively. In contrast, with adaptation, the gamma index (2%, 2 mm) was 94% and 96%, respectively.

## 4. Discussion

We have demonstrated the feasibility and dosimetric accuracy of the simple RGAPT strategy in a way like unit testing for each stage illustrated in figure 1. It is important to note that any uncertainties at each stage would propagate to the subsequent one in a sequential processing manner.

We tested the range measurability in 60 s of PET acquisition. All three types of probing beams, one, three, and five layers, were able to generate PET images that showed activity range. The single-layer mid-range probing with 1.65 MU did not provide clear separations of activity range for different range shift scenarios, while both the three-layer (5.55 MU) and five-layer (8.71 MU) probing provided accurate range measurements with an uncertainty of 0.5 mm. In the test, the range shift was measured against the ground truth. In a realistic situation, where the ground truth is unavailable, the range shift would be measured against the expected range obtained via MC simulations, potentially introducing additional uncertainty.

In the film verification, the single-layer probing did not sufficiently expose the film for dose conversion though the raw intensity could still be used to distinguish the range shifts. All three probing beam selections yielded similar range shifts, consistent with the expected shifts from changes in the WEPL, as confirmed by film measurements. However, in the overshoot scenario created by removing the Lucite sheets, the resulting BR shifts were slightly larger than the changes in WEPL, possibly attributable to range straggling. In realistic patients’ cases, heterogeneous tissues may induce more range straggling. However, this effect might not compromise range measurement accuracy, as the measurements are compared with calculations that can incorporate heterogeneity and model range straggling via simulation.

In addition to comparing probing beam dose, films were also used to compare plans. Without adaptation, the gamma (2%, 2 mm) passing rates for overshoot and undershoot were 72% and 66% (average 69%), respectively. In contrast, with adaptation, the gamma (2%, 2 mm) passing rates were 94% and 96% (average 95%), respectively. The film measurements as implemented were not ideal for absolute dosimetry, which may require ion chamber measurements, but serve well for relative dosimetry and profile comparison as they are commonly used in photon beams.

We only tested the placements of 0, 4, and 8 Lucite sheets, enabling the study of ±6.8 mm range shifts, as we were initially uncertain about the range of measurement uncertainty. In the experiment, we found that both PET and film measurements could achieve sub-millimeter accuracy. These results can be considered as a sanity check of the RGAPT process, demonstrating the feasibility of mid-range probing, and serve as the foundation for setting up more challenging cases in the future.

Ideally, the probing spots should cover the cross-section of the treatment beam to enable the detection of range shifts along different paths of the beam direction. In the test, the midrange layers did cover the cross-section, albeit with varying spot intensities. This did not pose an issue, as the range shifts were homogeneous in the test. In realistic scenarios where inhomogeneous range shifts may occur, selecting probing spots should account for individual spot intensity to ensure the measurability of range shifts across the cross-section.

The simple adaptation strategy yielded dose very similar to the plan with a high gamma passing rate though there could be small deviations of spot intensity from the reference plan in a few middle layers (up to five layers in the experiment) due to the non-negativity criteria. Both being small and in the less dominant layers (middle layers as opposed to distal layers) helped to reduce the impact of spot intensity deviations on the dose.

We only experimented with single fields and simple target geometry, which made it easier to control experimental scenarios and measure the BR shift than using complex plans. In addition, it also made clearer the effect of adaptive dose with single fields than multiple fields since in the latter case dose could be smeared. Nevertheless, realistic plans, such as clinical plans, are necessary for clinical feasibility, while the single field experiments could lay a good basis moving forward.

Though any range changes after the midrange spots are not captured by midrange probing, this typically does not affect the dose unless the changes occur within the tumor region between the midrange and the distal layer. If the changes are due to tumor deformation according to the online images, recontouring of the tumor may be necessary, followed by treatment plan re-optimization.

Potentially, instead of reconstructing the PET image of source distribution, we may study the activity range via line of response or time-of-flight analysis (Cheng *et al* 2022, Ma *et al* 2022), which may allow us to improve range measurement uncertainty.

Though proton planning affords a high degree of freedom, plan optimization often only uses a small subset of available variables limited by computational power. We previously proposed a global optimization framework that models spot position as a continuous variable, allowing for densely sampled spots (Chen *et al* 2022). The probing beam selection could be more flexible for such plans. For example, the probing beam intensity could be increased while still providing room for plan adaptation when dense spots are utilized and increase optimization capacity.

In vivo range measurements and adaptive planning are generally treated as research topics for separate problems. The former addresses the systematic uncertainty (error), such as Hounsfield calibration errors, and the latter addresses the random error, such as daily anatomical changes (Lomax 2016). Our RGAPT combined the in vivo and adaptive through a judicious use of treatment beams: delivering mid-range treatment beams as probing beams and measuring the BRs in vivo; and utilizing the in vivo measurements for online adaptive planning in both accurate and timely manners.

## 5. Conclusion

RGAPT planning and delivery are feasible as demonstrated by the experimental verification. Considering the BR measurement accuracy and the low number of mid-range layers, the three-layer mid-range spots were most suitable for range probing. The delivery workflow had a small increase in treatment time, ~1 min to wait for the in-beam PET data acquisition and ~1 min for image reconstruction. Uploading the adaptive plan had a minimal increase in time. The dosimetric accuracy of RGAPT was verified by film measurements with an average gamma (2%/2 mm) passing rate of 95%.

## Figures and Tables

**Figure 12.**
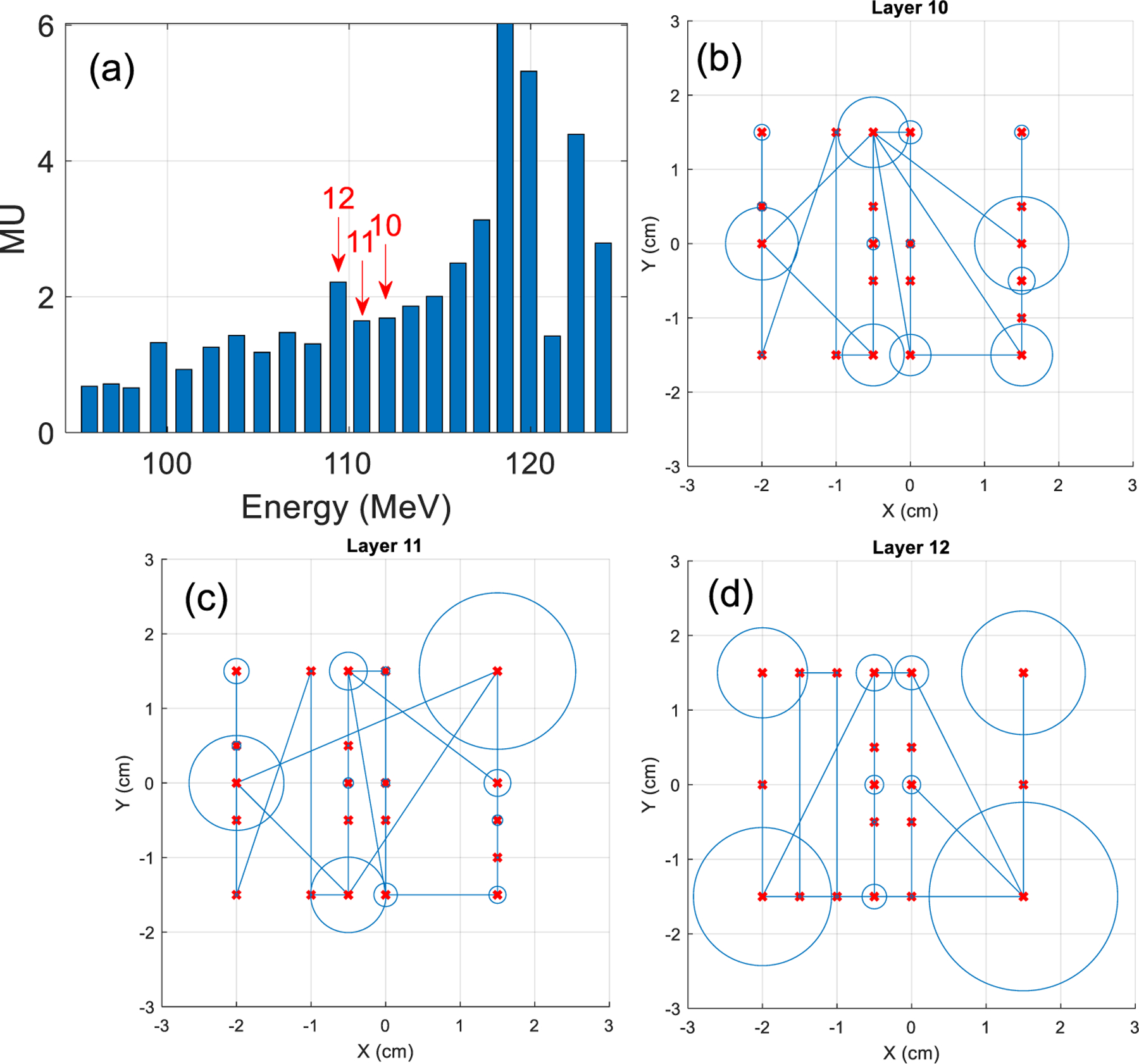
Illustration of (a) the MU distribution and (b)–(d) three mid-range layers: #10-#12, of the reference plan. In (a), the MU distribution is plotted against the energy layers, with three mid-range layers (#10-#12) indicated by red arrows. In (b)–(d), each midrange layer consists of multiple spots, with the spot center position indicated by red ‘x’ and spot intensity indicated by the circle size. The spots are connected according to the delivery sequence.

**Table 1.** Probing beam profile data summary.

Mid-range	Lucite	*D*_max_ (cGy)	*R*50, Δ (mm)
3-layer	4	20.6	38.6
	0	20.6	+7.3
	8	19.6	−6.8
5-layer	4	28.3	39.2
	0	30.3	+7.3
	8	30.8	−6.6

**Table 2.** *γ* passing rates comparing non-adaptive plans to the reference.

*γ*	Non-adaptive (0 sheet) vs Ref			Non-adaptive (8 sheet) vs Ref		
Criteria	1%	2%	3%	1%	2%	3%
1 mm	54.52	58.05	61.78	51.92	54.34	57.01
2 mm	70.02	72.11	74.31	64.62	65.88	67.22
3 mm	78.10	79.68	81.32	71.41	72.28	73.22

**Table 3.** *γ* passing rates comparing adaptive plans to the reference.

*γ*	Adaptive (0 sheet) vs Ref			Adaptive (8 sheet) vs Ref		
Criteria	1%	2%	3%	1%	2%	3%
1 mm	63.75	68.32	73.29	82.99	87.13	91.02
2 mm	92.67	94.18	95.76	94.34	96.08	97.44
3 mm	97.35	98.10	98.67	97.79	98.47	99.02

## Data Availability

The data cannot be made publicly available upon publication because they are not available in a format that is sufficiently accessible or reusable by other researchers. The data that support the findings of this study are available upon reasonable request from the authors.

## Appendix

The reference treatment plan created by the Eclipse TPS consisted of 22 layers and 1382 spots. In figure 12, we depict its MU distribution and mid-range layers. Figure 12(a) shows the MU distribution across different energy layers. The layer numbers are listed in the order of decreasing energy, aligning with the delivery control point sequence from high to low energy. This distribution illustrates that the MU increases towards higher energy, consistent with the principle of utilizing spread-out Bragg peaks for SFUD delivery. Figures 12(b)–(d) depict three mid-range layers, #10-#12, respectively, with a total of 5.5 MU. In each layer, the spot centers are shown in the red ‘x’ and connected by the delivery path, and the circle size indicates the spot weight. Note that a spot location may count as multiple spots due to repeated hits during spot scanning.
